# Salivary IL-17 and IL-10 as Potential Diagnostic Biomarkers of Different Stages of Periodontitis in Smoker and Nonsmoker Patients

**DOI:** 10.1055/s-0043-1768154

**Published:** 2023-05-12

**Authors:** Maha Abdulsalam Mohammed, Raghad Fadhil Abbas, Hadeel Mazin Akram

**Affiliations:** 1 Department of Periodontology, College of Dentistry, University of Baghdad, Baghdad, Iraq

**Keywords:** IL-10, IL-17, periodontitis, diagnosis

## Abstract

**Objectives** The gold standard in the field of periodontal research currently is to find a valid biomarker that can reliably be used for diagnosing periodontal diseases. Given the limitations of the current diagnostic tools that stall to predict susceptible individuals and determine whether active tissue destruction is occurring, there is an increased urge to develop alternative diagnostic techniques that would compensate for the problems inherited in these available methods, such as measuring levels of biomarkers present in oral fluids such as saliva; so the aim of this study was to determine the diagnostic potential of interleukin-17 (IL-17) and IL-10 to differentiate periodontal health from smoker and nonsmoker periodontitis, and to differentiate among different stages (severities) of periodontitis.

**Materials and Methods** An observational case–control study was performed on 175 systemically healthy participants grouped into healthy as controls and periodontitis as cases. Periodontitis cases were divided according to the severity into stages I, II, and III, and each of the stages was further subdivided into smokers and nonsmokers patients. Unstimulated saliva samples were collected, clinical parameters were recorded, and salivary levels were assayed using enzyme-linked immunosorbent assay.

**Results** Elevated levels of IL-17 and IL-10 were associated with stage I and II compared with the healthy controls. However, a significant decrease in stage III was observed compared with the control group for both biomarkers.

**Conclusion** Salivary IL-17 and IL-10 might be useful for distinguishing periodontal health from periodontitis; however, further research is needed to substantiate their use as potential biomarkers for the diagnosis of periodontitis

## Introduction

Periodontitis can be defined as an inflammatory disorder that causes tissue and bone loss as a consequence of various interactions between the host immune response and pathogenic bacteria. 1 These interactions are modified by several genetic and environmental factors together with the presence of systemic diseases and habits such as smoking. 2

Knowledge of how immune mechanisms—inflammatory responses—are regulated is critical for understanding the pathogenesis of complex diseases, such as periodontitis. 3 As part of the immune system, cytokines and chemokine signals regulate the immune response to infection. Cytokines are highly important peptide mediators responsible for cell signaling and communication. Functions of cytokines vary to include the control of cell proliferation, cell differentiation, immune responses, and inflammatory responses. 4 In periodontal diseases, the balance between the proinflammatory and anti-inflammatory cytokines is generally tipped toward the proinflammatory activity. 5 Among the proinflammatory cytokines is interleukin-17 (IL-17); this cytokine appears to be of particular interest in the pathogenesis of periodontitis because of its involvement in both inflammation and protective antimicrobial immunity. 6 Another host-derived mediator is IL-10, which possesses a potent anti-inflammatory activity to suppress the expression of several proinflammatory mediators such as tumor necrosis factor-α, IL-6, or IL-1. 7

Risk factors are part of the causal chain for a particular disease or can lead to an individual's exposure to disease. For periodontal disease, smoking, in particular, has a consistent, positive association with loss of periodontal attachment, which is confirmed in many studies. 8

Despite the improvements in the classification system since the introduction of the 2017 classification of periodontal and peri-implant diseases and conditions, the problems associated with the existing clinical diagnostic methods (inspection, palpation, periodontal probing, and radiography) are still present such as sensitivity of probing technique to the applied force, dimensions of the probe, and the limitations of conventional radiograph. 9 This has increased the urge to develop a more sophisticated and precise predictors for periodontal disease; one of them is using biomarkers present in oral fluids such as Gingival crevicular fluid (GCF) and saliva.

Keeping these observations in mind, rapid chair-side tests, which rely on biomarkers present in biological fluids, are developed to diagnose periodontal disease, and are called point of care (POC) diagnostics that simplifies diagnosis and helps improve the prognosis. The use of saliva in (POC) diagnostics offers many advantages as it is readily available, and contains a rich array of diagnostic biomarker molecules with the ability to obtain rapid and reliable results. 10 Based on the facts mentioned above, detecting a biomarker that can reliably be used to establish an accurate periodontitis diagnosis has become pivotal.

## Materials and Methods

An observational case–control study is a design that was applied to this study. The potential patients were recruited from individuals seeking periodontal therapy at the College of dentistry hospital/the University of Baghdad. The study started in January 2022 and finished in August 2022. Each patient was requested to sign an informed consent form after providing all the information describing the study's aim. This study was also conducted following ethical principles, including the World Medical Association Declaration of Helsinki.

### Sample Size and Study Population

The sample size was calculated by using one of the biomarkers (IL-17) as the study's primary outcome. The concentration of this biomarker during health was estimated to be equal to 4.29 pg/mL, whereas, during periodontitis, its concentration was regulated as up to 12.68 pg/mL. 11 This yields an expected odds ratio of 3 between periodontal health and periodontitis, which was used to calculate sample size using online tool ( https://epitools.ausvet.com.au/samplesize ) at a 95% confidence interval and 5% error margin. The estimated sample size for the periodontitis group is 106, rounded up to 150 to avoid drop out of the sample and attrition bias, while it was 25 for healthy control. Accordingly, each periodontitis group (stage I, II, III) with and without smoking received around 50 patients, following allocation ratio of 1:2:2:2(control: smoker and non-smoker periodontitis stage I: smoker and non-smoker periodontitis stage II: smoker and non-smoker periodontitis stage III, respectively).

The 175 systemically healthy subjects were divided into four groups:

- Periodontally healthy with intact periodontium as the control group.
- Periodontitis stage I (bone loss involving 1–2mm of the root).
- Periodontitis stage II (bone loss involves the coronal ⅓ of the root).
- Periodontitis stage III (bone loss extends to the middle ⅓ of the root).

Periodontal health was defined by the presence of pocket probing depth (PPD) less than or equal to 3mm, bleeding on probing less than 10% and no clinical attachment loss (CAL) due to periodontitis. 1

However, all periodontitis cases exhibited generalized form (≥30% of teeth involved) and unstable status (PPD ≥5mm or PPD 4mm with bleeding on probing [BOP]). 12

Then each of the three periodontitis group was subcategorized into two group:

- Smokers (stage I, II, III)
- Non-smokers (stage I, II, III)

The following data were obtained from subjects belonging to the smokers' group: (1) number of cigarettes consumed daily, (2) frequency of smoking, and (3) number of years of smoking. The criteria for smoking status included in this study were applied according to Centers for Disease Control and Prevention (CDC) heavier smokers, defined as those who smoked more than 16 cigarettes per day within the past 30 days. 13

### Eligibility Criteria

#### Inclusion Criteria

Systemically healthy patients (excluding the case definition criteria) eligible to be included in the study have a minimum of 20 teeth.

#### Exclusion Criteria

Patients with medical disorders such as diabetes mellitus, immunologic diseases and hepatitis, and those who had received antibiotic or periodontal treatment in the previous 3 months, diabetes mellitus, previous history of organ transplant or cancer therapy, or had any cardiovascular disease excluded. Additional exclusion criteria include obese patients with body mass index (BMI) more than or equal to 30.

### Clinical Findings

A complete mouth examination was performed using a periodontal probe (UCN-15 probe) by a calibrated periodontist. Clinical periodontal recordings were performed on all dentition, including dichotomous plaque index (PLI) (±) by using disclosing agents, 14 bleeding on probing (BOP %), pocket probing depth (PPD), and CAL. BMI index was also added to the above clinical measurements to exclude obese patients. The third molars were also excluded.

### Salivary Collection Procedure

Unstimulated saliva samples were collected from all patients before clinical evaluation; the patients were instructed not to consume any food or drink at least 1 hour before the saliva collection. And while they were sitting straight and their heads bent forward, the saliva was emptied from the bottom lip into a plastic cup during a 5-minute period. Then, the total saliva collected was aspirated from the disposable cup using a micropipette to aspirate a measured volume of the saliva of 500μl into a plastic Eppendorf tube. After collection, samples were centrifuged at 3000 rpm for 10 minutes to separate the cellular debris from the salivary supernatants. After being centrifuged and separated from the cellular debris, the salivary fluid was aspirated again, stored in a clean and labeled Eppendorf tube, and frozen at -40°C until the day of analysis by enzyme-linked immunosorbent assay (ELISA) kits for IL-10 and IL-17.

### Enzyme-Linked Immunosorbent Assay

Salivary samples were added in triplicate to the wells of microtiter plates to determine the concentrations of human IL-17 and IL-10 using Shanghai YL Biotech ELISA Kits. The IL-10 and IL-17 levels were calculated from the standard curves included in each assay. The levels in the saliva were expressed as ng/mL.

### Statistical Analysis

Data description, analysis, and presentation were performed using Statistical Package for Social Science (SPSS version 21). The Shapiro–Wilk test was used to check the distribution of data that indicated normal distribution; thus, multigroup comparisons were conducted using the analysis of variance test. In case of significance, additional intergroup comparisons were performed using Bonferroni posthoc test. The diagnostic accuracy of the biomarkers was determined using ROC (receiver operating characteristic) and AUC (area under curve). A level of the *p* -value of less than 0.05 was considered statistically significant.

## Results

### Demographic Characteristics

The highest mean of age was seen in stage III smokers (46.280), while the youngest were in the control group (37.440), with no significant differences ( Table 1 ). Additionally, gender distribution ( Table 2 ) also showed no significant differences among the groups.

**Table 1:** Descriptive and statistical test of age (years) among groups

**Stages**	**Groups**	**Mean (y)**	** ± SD**	** ± SE**	**Min. years**	**Max. years**	**F**	***p*** **-Value**
	Control	37.440	10.239	2.048	20.000	55.000	2.019	0.066 NS
Stage I	Nonsmoker	39.640	9.630	1.926	22.000	55.000		
	Smoker	40.680	8.669	1.734	27.000	55.000		
Stage II	Nonsmoker	40.720	8.820	1.764	27.000	58.000		
	Smoker	43.120	11.487	2.297	20.000	65.000		
Stage III	Nonsmoker	41.640	10.527	2.105	28.000	63.000		
	Smoker	46.280	8.928	1.786	20.000	62.000		

Abbreviations: F, F test; Max, maximum; Min, minimum; NS, nonsignificant; SD, standard deviation; SE, standard error.

**Table 2:** Association between gender and groups

			Stage I		Stage II		Stage III				Total
		Control	Nonsmoker	Smoker	Nonsmoker	Smoker	Nonsmoker	Smoker	Chi-squared test	*p* -Value	
M	N.	15	18	10	10	10	10	10	10.405	0.109 NS	83
	%	60	72	40	40	40	40	40			47.43
F	N.	10	7	15	15	15	15	15			92
	%	40	28	60	60	60	60	60			52.57

Abbreviations: F, female; M, male; N, number; NS, nonsignificant.

### Clinical Periodontal Parameters

**Fig. 1:**
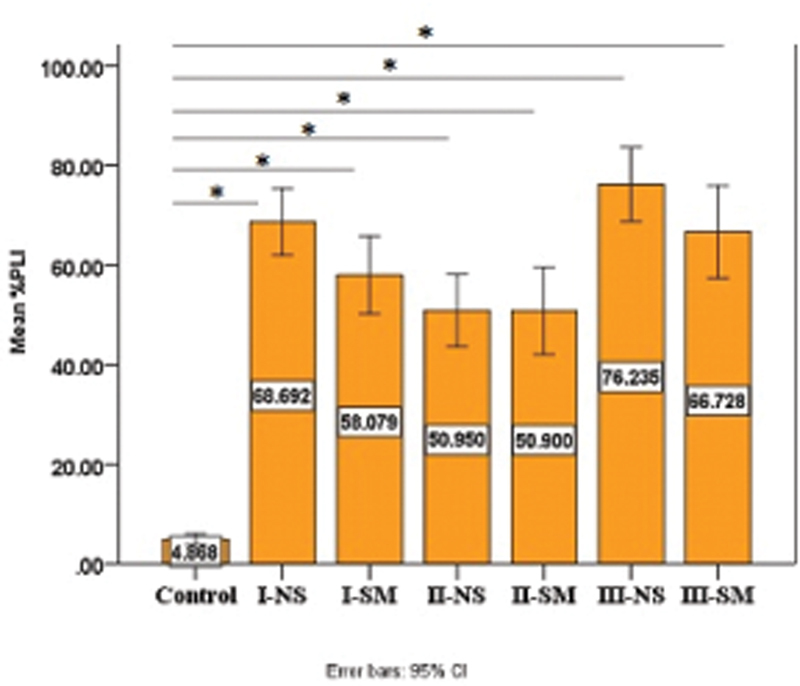
Comparison of mean percentage of plaque index (PLI) among all groups. Periodontitis (stage I–III) smoker (SM) and nonsmoker (NS) groups exhibited significantly higher percentage of PLI than control group. BOP, bleeding on probing, * *p* <0.05. CI, confidence interval.

**Fig. 2:**
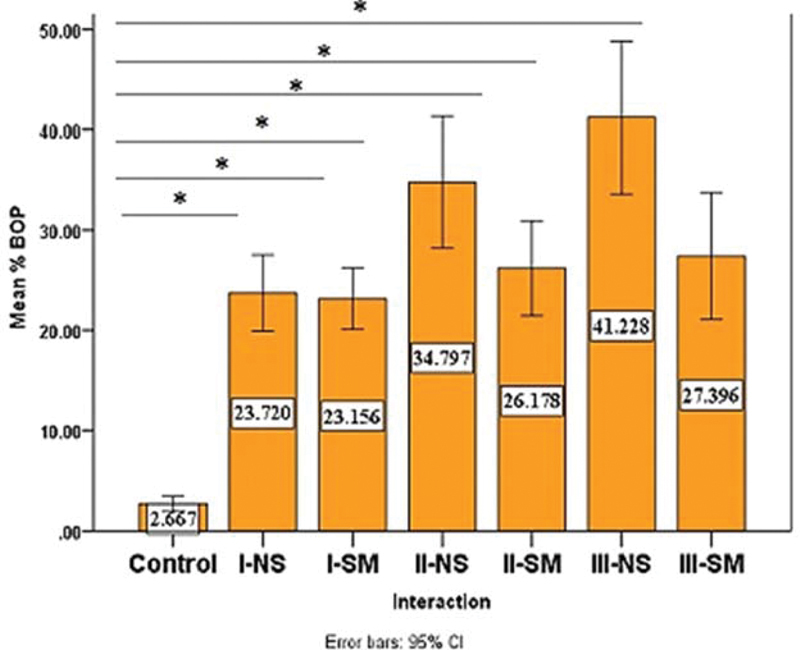
Comparison of mean percentage of bleeding on probing (BOP) among groups, all periodontitis groups exhibited significantly greater mean percentage of BOP compared with the control group. Additionally, mean percentage of BOP was lower for the smoker's group than non-smokers for all three stages of periodontitis, * *p* < 0.05. CI, confidence interval.

Clinical periodontal parameters in terms of the PLI ( Fig. 1 ) and BOP ( Fig. 2 ) scores were significantly higher in all case groups compared with the control group ( *p* -value= 0.00000) for both indicators.

The mean of BOP% in the smokers' group was significantly lower than that of the nonsmokers in both stages II ( *p* -value= 0.026) and III ( *p* -value= 0.000; Table 3 ).

**Table 3:** Descriptive and statistical test of BOP percentage among groups

**Groups**	**Control**	**Mean**	** ± SD**	** ± SE**	**Minimum**	**Maximum**	**F**	***p*** **-Value**
		2.667	1.743	0.349	0.595	6.548		
Stage I nonsmoker		23.720	9.276	1.855	10.714	59.333	0.022	0.883
Stage I smoker		23.156	7.353	1.471	11.333	39.744		
Stage II nonsmoker		34.797	15.923	3.185	17.164	78.030	5.062	0.026
Stage II smoker		26.178	11.453	2.291	13.043	66.000		
Stage III nonsmoker		41.228	18.462	3.692	14.198	79.710	13.039	0.000
Stage III smoker		27.396	15.324	3.065	5.952	70.238		
Nonsmoker stages	F	10.690						
	*p* -Value	0.000047						
Smoker stages	F	0.650						
	*p* -Value	0.524						

Abbreviations: BOP, bleeding on probing; F, F test; Max, maximum; Min, minimum; SD, standard deviation; SE, standard error.

**Fig. 3:**
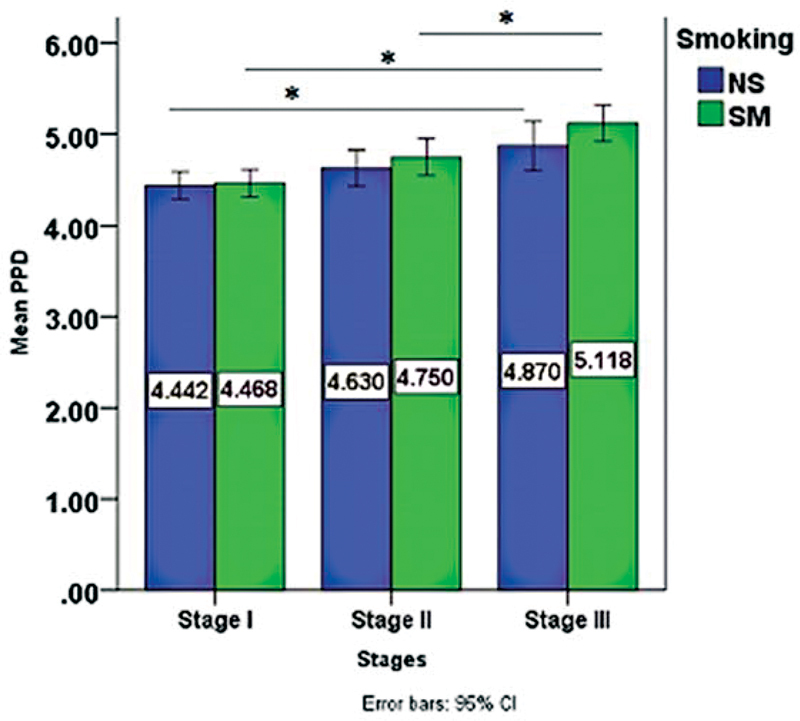
Comparison of the mean PPD scores among periodontitis stage I, II, III. Periodontitis stage III exhibited the highest PPD mean compared with stage II and I. NS, nonsmoker; PPD, pocket probing depth; SM, smoker, * *p* < 0.05. CI, confidence interval.

**Fig. 4:**
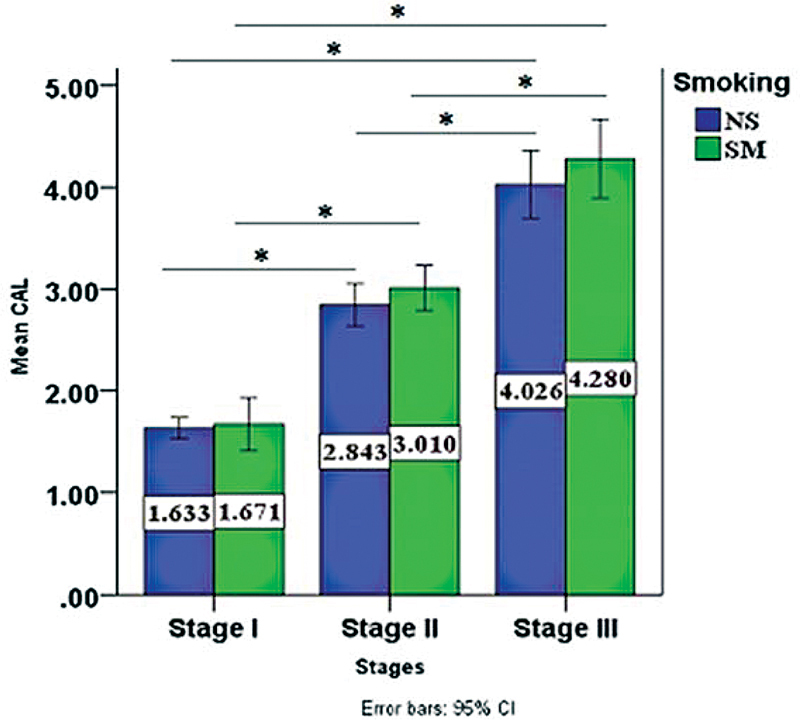
Comparison of mean clinical attachment loss (CAL) among periodontitis stage I, II, III, stage III group exhibited significantly higher CAL than stage II and I. CI, confidence interval; NS, nonsmoker; SM, smoker.

As far as PPD and CAL are concerned, both were highest in stage III smokers' group, as seen in Figs. 3 and 4 , respectively, with PPD having a statistical significance between stage I and III ( *p* -value= 0.006) only among the nonsmokers' periodontitis stages, while in the smoker's periodontitis stages a significant difference was found between stage I and III ( *p* -value= 0.000), stage II and III as well ( *p* -value= 0.022).

### Salivary Biomarkers Levels

**Fig. 5:**
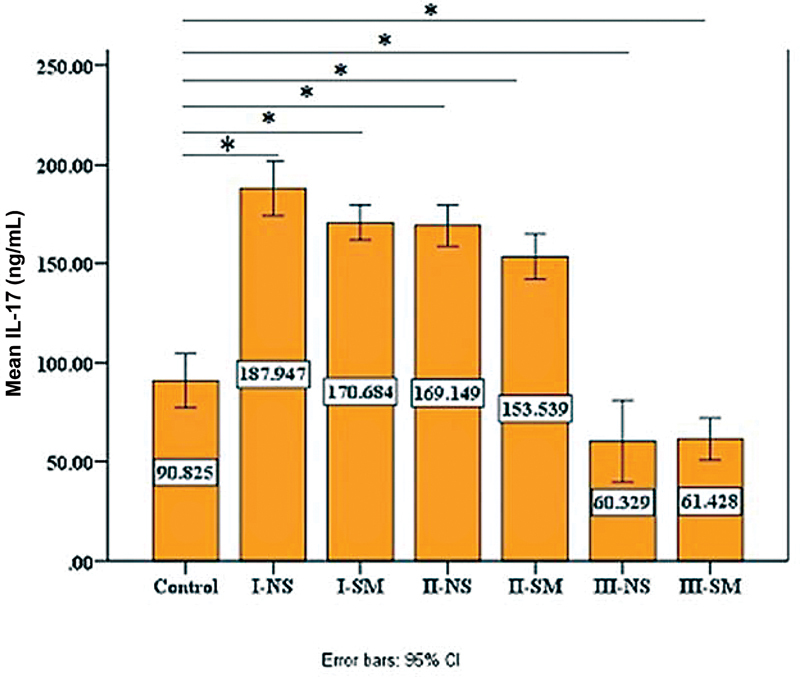
Comparison of mean interleukin-17 (IL-17) levels among groups, the highest mean seen in stage I nonsmokers (NS) group and the lowest in stage III smokers' (SM) group. * *p* < 0.05. CI, confidence interval.

**Fig. 6:**
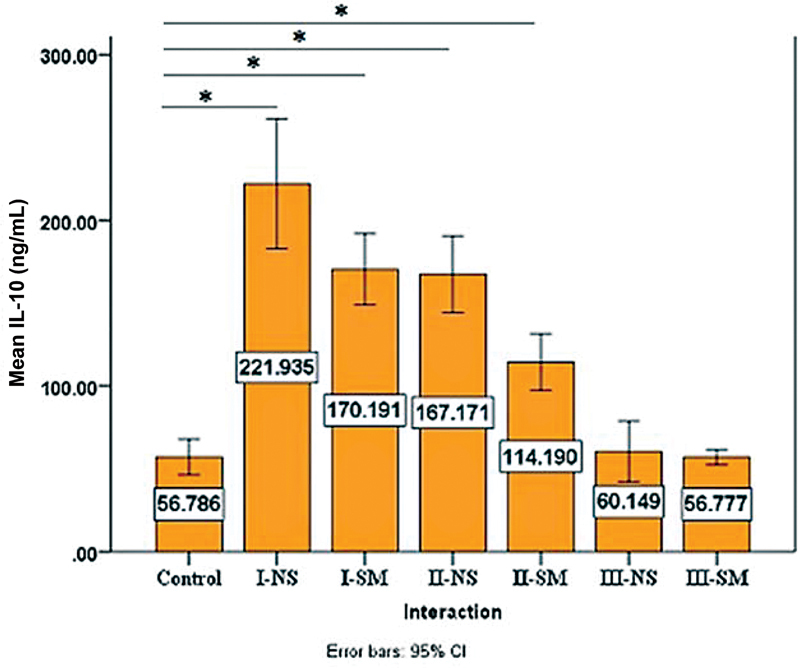
Comparing the mean levels of interleukin-10 (IL-10) among all study groups, the highest mean was associated with stage I nonsmokers (NS) and the lowest seen in stage III smokers (SM). CI, confidence interval * *p* <0.05.

The results showed a statistically significant difference, with higher levels of the biomarkers in all periodontitis groups compared with controls except for stage III, where the levels of IL-17 dropped significantly ( Table 4 , Fig. 5 ), while for IL-10, there showed to be a difference between stage III and the controls; however, it is of no statistical significance ( Table 5 , Fig. 6 ).

**Table 4:** Comparison of IL-17 of each group with control using Dunnett two-sided

F	*p* -Value	(I) Interaction	(J) Interaction	Mean difference (I-J)	*p* -Value
73.207	0.000	Stage I nonsmoker	Control	97.122	0.00000
		Stage I smoker	Control	79.859	0.00000
		Stage II nonsmoker	Control	78.324	0.00000
		Stage II smoker	Control	62.714	0.00000
		Stage III nonsmoker	Control	− 30.496-	0.00534
		Stage III smoker	Control	− 29.398-	0.00789

Abbreviation: IL-17, interleukin-17.

**Table 5:** Comparisons of IL-10 of each group with control using Dunnett two-sided

F	*p* -Value	(I) Interaction	(J) Interaction	Mean difference (I-J)	*p* -Value
40.075	0.000	Stage I non smoker	Control	165.149	0.000
		Stage I smoker	Control	113.405	0.000
		Stage II nonsmoker	Control	110.385	0.000
		Stage II smoker	Control	57.404	0.001
		Stage III nonsmoker	Control	3.363	1.000
		Stage III smoker	Control	− 0.009	1.000

Abbreviation: IL-10, interleukin-10.

For IL-17, there was no difference between the smokers and nonsmokers periodontitis of each stage ( Table 6 ); meanwhile, a statistically significant difference between stage I and III, stage II and III ( *p* -value= 0.000) in both smoker's periodontitis stages and nonsmokers ( Table 7 ). On the contrary, IL-10 shows a significant difference between the smokers and nonsmokers of stages I and II ( *p* -value= 0.001; Table 8 ); additionally, a statistical significance was found among stages of periodontitis in both smokers and nonsmokers detailed in Table 9 .

**Table 6:** Descriptive and statistical test of IL-17 means among groups

**Groups**	**Control**	**Mean**	**± SD**	**± SE**	**Minimum**	**Maximum**	**F**	***p*** **-Value**
		90.825	32.861	6.572	33.047	158.221		
Stage I nonsmoker		187.947	33.514	6.703	129.218	287.766	3.633	0.059
Stage I smoker		170.684	21.438	4.288	131.961	212.276		
Stage II nonsmoker		169.149	25.211	5.042	100.262	228.970	2.970	0.087
Stage II smoker		153.539	27.646	5.529	86.657	193.963		
Stage III nonsmoker		60.329	50.138	10.028	11.124	192.174	0.015	0.904
Stage III smoker		61.428	25.620	5.124	19.672	100.607		
Nonsmoker stages	F	115.726						
	*p* -Value	0.000						
smoker stages	F	84.171						
	*p* -Value	0.000						

Abbreviations: F, F test; IL-17, interleukin-17; SD, standard deviation; SE, standard error.

**Table 7:** Multiple pairwise comparisons of IL-17 among stages using Bonferroni test

Smoking	Stage	Stage	Mean difference	*p* -Value
Nonsmoker	Stage I	Stage II	18.798	0.119
		Stage III	127.619	0.000
	Stage II	Stage III	108.820	0.000
Smoker	Stage I	Stage II	17.144	0.181
		Stage III	109.256	0.000
	Stage II	Stage III	92.112	0.000

Abbreviation: IL-17, interleukin-17.

**Table 8:** Descriptive and statistical test of IL-10 among groups

**Groups**	**Control**	**Mean**	** ± SD**	** ± SE**	**Minimum**	**Maximum**	**F**	***p*** **-Value**
		56.786	25.882	5.176	17.441	124.696		
Stage I nonsmoker		221.935	95.335	19.067	48.145	399.261	10.688	0.001
Stage I smoker		170.191	52.276	10.455	77.312	277.898		
Stage II nonsmoker		167.171	56.388	11.278	62.664	245.365	11.205	0.001
Stage II smoker		114.190	41.345	8.269	58.305	196.250		
Stage III nonsmoker		60.149	44.209	8.842	20.835	230.306	0.045	0.832
Stage III smoker		56.777	11.135	2.227	42.271	78.910		
Nonsmoker stages	F	54.058						
	*p* -Value	0.000						
Smoker stages	F	25.674						
	*p* -Value	0.000						

Abbreviations: F, F test; IL-10, interleukin-10; SD, standard deviation; SE, standard error.

**Table 9:** Multiple pairwise comparison of IL-10 among stages by groups using Bonferroni posthoc test

Smoking	Stage	Stage	Mean difference	*p* -Value
Nonsmoker	Stage I	Stage II	54.765	0.002
		Stage III	161.786	0.000
	Stage II	Stage III	107.022	0.000
Smoker	Stage I	Stage II	56.001	0.002
		Stage III	113.414	0.000
	Stage II	Stage III	57.413	0.001

Abbreviation: IL-10, interleukin-10.

Finally, the findings in Table 10 explain how the two biomarkers are correlated to one another.

**Table 10:** Correlation between IL-10 and IL-17

**Interaction**		**IL-10**	
		*r*	*p* -Value
Control	IL-17	−0.428	0.033
Stage I nonsmoker	IL-17	0.040	0.850
Stage I smoker	IL-17	−0.270	0.192
Stage II nonsmoker	IL-17	−0.070	0.740
Stage II smoker	IL-17	−0.460	0.021
Stage III nonsmoker	IL-17	0.613	0.001
Stage III smoker	IL-17	−0.162	0.439

Abbreviation: IL-17, interleukin-17.

There seems to be a significantly negative correlation in the controls group ( *p* -value= 0.033), and stage II smoker's group ( *p* -value= 0.021).

The biomarkers correlated significantly and positively to each other only in nonsmoker's group of stage III ( *p* -value= 0.001)

### Diagnostic Accuracy of IL-17 and IL-10 in Discriminating Periodontal Health and Disease

**Fig. 7:**
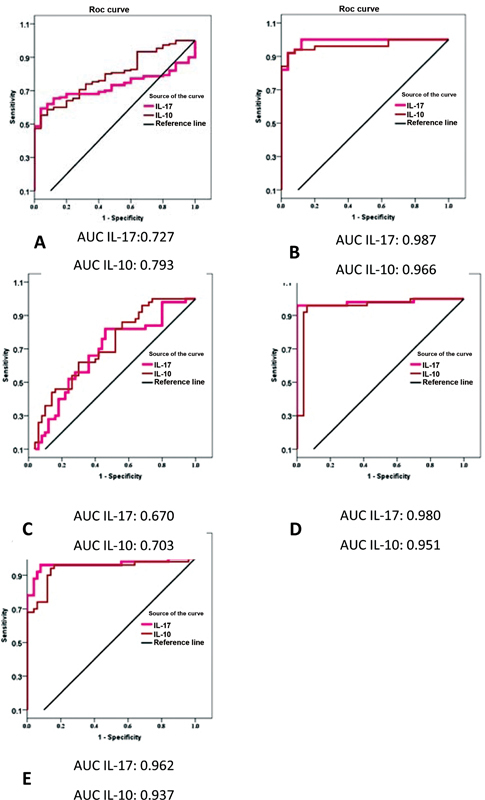
Receiver operating characteristic (ROC) curves of interleukin-17 (IL-17) and IL-10. ( **A** ) Periodontitis versus healthy control. ( **B** ) Stage I versus healthy control, ( **C** ) stage I versus stage II, ( **D** ) stage I versus stage III, and ( **E** ) stage II versus stage III. AUC, area under curve.

Diagnostic accuracy was determined by using ROC to evaluate the sensitivity and specificity of each biomarker to differentiate periodontal health from periodontitis and between the different stages of periodontitis. Fig. 7 shows that AUC for salivary IL-17 and IL-10 was 0.727 and 0.793, respectively, suggesting a potential to discriminate between periodontal health and periodontitis. Additionally, the above-stated biomarkers showed high diagnostic accuracy in differentiating the different stages of periodontitis.

Generally, IL-17 showed a higher degree of sensitivity and specificity than IL-10, with the highest observed association with IL-17 at a cutoff point of 127.985 ( Table 11 ).

**Table 11:** ROC between control and periodontitis and among different stages of periodontitis

	Test result variable(s)	AUC		*p* -Value	Optimal cutoff point	%Sensitivity	%Specificity
PD X control	IL-17	0.727	Good	0.000	128.164	65.3	88
	IL-10	0.793	Good	0.000	63.1120	70.7	72
Stage I X control	IL-17	0.987	Excellent	0.000	139.728	92	96
	IL-10	0.966	Excellent	0.000	94.732	92	96
Stage I–stage II	IL-17	0.670	Sufficient	0.003	178.564	82	54
	IL-10	0.703	Good	0.000	151.867	62	70
Stage I–stage III	IL-17	0.980	Excellent	0.000	127.985	96	100
	IL-10	0.951	Excellent	0.000	77.26	92	96
Stage II–stage III	IL-17	0.962	Excellent	0.000	119.547	92	94
	IL-10	0.937	Excellent	0.000	78.563	94	86

Abbreviations: AUC, area under curve; IL-17, interleukin-17; ROC, receiver operating characteristic.

**Fig. 8:**
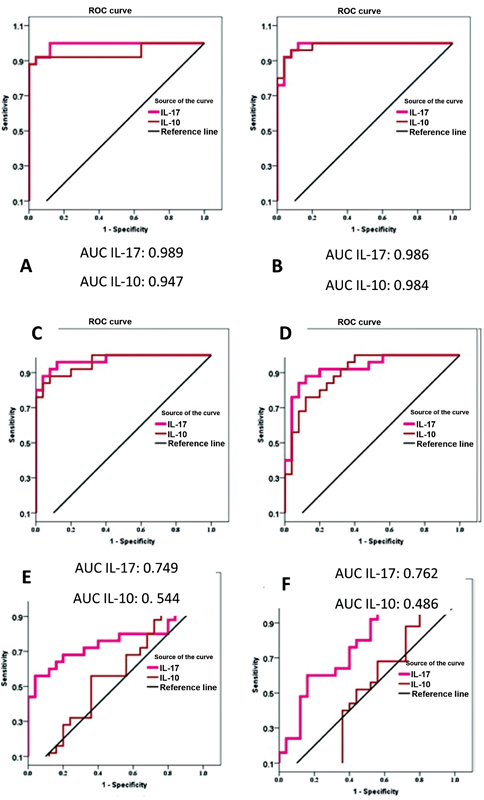
Receiver operating characteristic (ROC) curves of interleukin-17 (IL-17) and IL-10, ( **A** ) stage I nonsmoker versus control, ( **B** ) stage I smoker versus control, ( **C** ) stage II nonsmoker versus control, ( **D** ) stage II smoker versus control, ( **E** ) stage III nonsmoker versus control, and ( **F** ) stage III smoker versus control. AUC, area under curve.

The ROC analysis for determining the diagnostic potential of the two biomarkers in differentiating each group of periodontitis from stage I to III smokers and nonsmokers from the healthy controls is illustrated in Fig. 8 . The biomarkers have shown good-to-excellent AUC values in discriminating between all smokers and non-smokers periodontitis stages from periodontal health ( Table 12 ), except for IL-10, which failed to distinguish the smokers and nonsmokers of stage III from the healthy controls.

**Table 12:** ROC of each group from control

	Test result variable(s)	AUC		*p* -Value	Optimal cut off point	%Sensitivity	%Specificity
Stage I nonsmokers	IL-17	0.989	Excellent	0.000	144.82	92	96
	IL-10	0.947	Excellent	0.000	106.345	92	96
Stage I smokers	IL-17	0.986	Excellent	0.000	134.101	92	96
	IL-10	0.984	Excellent	0.000	94.372	92	96
Stage II nonsmokers	IL-17	0.973	Excellent	0.000	134.715	92	92
	IL-10	0.960	Excellent	0.000	91.502	88	92
Stage II smokers	IL-17	0.925	Excellent	0.000	129.84	88	88
	IL-10	0.899	Very good	0.000	89.158	76	88
Stage III nonsmokers	IL-17	0.749	Good	0.003	66.67	68	80
	IL-10	0.544	Bad	0.594	50.613	56	64
Stage III smokers	IL-17	0.762	Good	0.002	63.995	60	84
	IL-10	0.486	Not useful	0.869	67.835	68	44

Abbreviations: AUC, area under curve; IL-17, interleukin-17; ROC, receiver operating characteristic.

## Discussion

This study was designed to determine the potential of IL-17 and IL-10 to diagnose patients with periodontitis by measuring their concentrations in saliva and also individuals with healthy gingiva along with the presence of smoking as a risk factor.

Increased level of PLI among all periodontitis stages was noted, which comes in accordance with Asif et al 15 who reported increasing plaque scores from mild, moderate-to-severe periodontitis; this is justifiable by the dose–response relationship between oral hygiene (OH) and periodontitis, as poorer OH results in higher plaque levels and thus, more periodontal destruction. 16

Also, smoking could exacerbate the condition as heat and accumulated product of combustion result in tobacco stain and calculus, which favor plaque accumulation. 17

With regard to BOP, it was highest in all periodontitis stages compared with controls reflecting the inflammatory state of tissues; this coincides with Hormdee et al 18 who reported similar findings.

The smokers presented a significant reduction in BOP% in each stage compared with nonsmokers; this coincides with Ali and Ali in 2012, 19 and this is probably due to tobacco smoking causing vasoconstriction of peripheral vessels. 20

Highest PPD belonged to stage III smokers with higher PPD values in the smoker's group of each stage, and this agrees with Velidandla et al, 21 who reported that cigarette smoking is associated with increased pocket severity, which is related to the local effect of smoke, altering the local temperature, and favoring plaque formation and, thus, more severe pocketing.

CAL was also shown to be higher in all smokers' periodontitis stages; this could be due to the change in the subgingival plaque composition, the virulence of subgingival bacteria, and alteration of the host response, which increase the destruction of periodontium and bone resorption, in addition to the damaging effects of nicotine in increasing the production of collagenase, suppressing the growth of gingival fibroblast, and the production of collagen and fibronectin. 22 These results were in corroboration with an overwhelming body of data from multiple studies that have demonstrated CAL is more prevalent and severe in patients who were tobacco users compared with nontobacco users. 23

Various reports show considerable variation in how IL-17 is expressed in periodontitis. IL-17 is a proinflammatory cytokine that serves the dual roles of protection and tissue destruction. On the protective side, IL-17 confers protective immunity against microbial pathogens by preserving barrier integrity and producing antimicrobial factors and granulocytes such as neutrophils and macrophages. 24 It can also promote the activation of osteoclasts and potentiate neutrophilic inflammation. 24 25 The above-stated properties can explain the upregulation of this cytokine observed in this study of periodontitis in stages I and II when compared with the healthy controls, similar to the reports by some authors regarding saliva, 11 26 GCF, 27 and serum, 28 which all stated an increase in IL-17 levels in periodontitis regardless of the differences in study settings.

It can be assumed that as the disease gets more advanced, higher proinflammatory interleukins concentration in saliva would be detected, but this is not the case in this study; the downregulation of IL-17 levels in saliva as periodontitis progressed to stage III was quite an exciting finding. A study conducted by Liukkonen et al demonstrated higher levels of IL-17 in localized periodontitis; meanwhile, these levels were significantly lower in healthy and in generalized periodontitis; he stated that in saliva, IL-17 concentrations increase at an early phase of periodontitis but then reduce when the disease progresses. 29 Sadeghi et al also reported lower IL-17 levels in GCF of periodontitis compared with those who were healthy and attributed it to the link that IL-17 has to bone resorption in periodontitis, so basically, its lower concentration in periodontally affected sites might be due to its consumption. 30

A recent study by Rodríguez-Montaño et al has reported a significant decrease in IL-17 levels in plasma of periodontitis, 31 and suggested the possibility that IL-17 is inversely proportional to the chronicity of the disease, a pattern that perhaps coincides with the results of the present study.

As far as smoking is concerned, it did not own a significant impact on IL-17 levels in each of the three stages of periodontitis; these results were in accordance with Sulistio et al, who reported no significant differences in total IL-17 levels in GCF between smokers and non-smokers with periodontitis 32 these results, however, fail to meet with results from Javed et al, who reported higher salivary IL-17 among cigarette smokers than nonsmokers with periodontitis. 33

Multiple possible explanations might justify these differences; one is that nonsmoker patients may have been passive smokers through exposure from individuals who actively smoke; this may affect the results since there is a piece of evidence that passive smoking can activate proinflammatory cytokines. 34 Additionally, in this study, heavy cigarette smokers were exclusively included based on CDC definition criteria, which might be different from the criteria used by other studies. Additionally, tobacco smoking was self-reported in this study. However, an accurate determination of a person being a smoker or a passive/nonsmoker can be done using an assessment of whole salivary cotinine levels. 35

Published literature on using salivary IL-17 as a diagnostic biomarker according to the 2017 classification for different stages of periodontitis is limited, and most of the available studies are comparative, only determined levels of IL-17 in health and disease.

This study, however, assessed the ability of this biomarker to diagnose periodontitis with different severities, and the results showed a potential of IL-17 to discriminate periodontal health and periodontitis with the highest AUC (0.987) between stage I and controls with 92% sensitivity and 96% specificity; also IL-17 was able to differentiate among the different stages of periodontitis; this coincides with Inönü et al who reported the potential of IL-17 to differentiate periodontal health from periodontitis with an AUC value of 0.807. 11 However, these findings were not consistent with a study by Ozçaka et al 36 who attributed the reduction of IL-17 levels in saliva to the possibility that saliva cannot reveal significant effects on IL-17 content, suggesting that it is useless for detecting disease presence and/or its severity. After all, the complex role of Th17 cells and its signature cytokine IL-17 in periodontitis, shown to be essential but is still controversial; many conflicting factors could have caused these variations. It could be the sampling technique, the different biological fluids, and tissues from which the samples were obtained, and also, what is worth mentioning is the state of periodontal disease activity (stability), namely periodontal tissue breakdown possibly being in the quiescent period when samples were collected.

IL-10 is the other diagnostic candidate of this study; this cytokine restricts and inhibits the action of multiple proinflammatory cytokines.

Significantly higher concentration of IL-10 in periodontitis stage I and II comparing it to health was noted; this comes in agreement with Fenol et al who found that IL-10 levels were higher in periodontitis than in health, attributing the results to the severity of the inflammatory process going on providing sufficient stimulus for a positive IL-10 response, 37 likewise, in a study by Varma et al, there was higher IL-10 levels in periodontitis stage I and II compared with health. 38 On the contrary, Tâlvan et al in 2017 39 reported higher levels of this cytokine in health despite differences in study design, samples, and settings, explained by its well-known role in maintaining the health and stability of periodontal tissues.

The result of this study also revealed decreasing levels of this biomarker along with the stages of periodontitis, and this is in solidity with Tâlvan et al in 2017, 39 where IL-10 levels decreased from early to generalized, being the lowest in aggressive periodontitis; thus, it is tempting to speculate that the higher expression of IL-10 accounts for the less severe form of the disease when compared with the progressed state of periodontitis.

Unlike IL-17, there is a downward trend of IL-10 levels with the potential to differentiate smokers from nonsmokers; similar findings were reported by He et al. 40 This might be explained by the tendency of smoking to exhibit suppressive action against anti-inflammatory molecules, including IL-10, which could be caused partly by the changes in vascular formations and microcirculatory functions in periodontal tissue due to smoking that can influence immune function and the subsequent inflammatory reaction in the gingiva. 41

From another perspective, some authors reported an IL-10 rise in smoker's periodontitis than nonsmokers, 42 and attributed it to the ability of smoking to disturb the balance between helper T cells toward a Th2 predominance and thus more of Th2 cytokines as IL-10.

As for the diagnostic potential of these biomarkers, currently, there is a limited number of published studies assessing the diagnostic potential of IL-10 for the staging of periodontitis to compare with.

In a study by Varma et al 2019, IL-10 has shown a significant difference between the health, gingivitis, and periodontitis group, but the difference between health and gingivitis was higher than that between health and periodontitis. 38

When correlating both biomarkers to each other, the correlation turned out to be negative, and this correlation seems logical as IL-17 is negatively regulated by several cytokines, one of which is IL-10. 43

Published literature has reported the antagonistic roles of these two cytokines with each other as Moretti et al and Sun et al 44 45 have described a dampening effect of IL-10 on the expression of IL-17 and indicating the protective role of IL-10 in suppressing an IL-17 periodontitis trait and the upregulation of IL-17 inflammatory responses in the condition of IL-10 deficiency.

When looking from a clinical point of view, the term “clinically significant” findings are those who make the patient improve the quality of life and makes him/her feel, function well and those which improve medical care. 46

This definition could be translated on findings of this study, meaning, if periodontitis could be diagnosed by a POC device using IL-17 or IL-10 levels in saliva, patients could easily diagnose their periodontitis at home and visit dental clinics at a suitable time; current disease activity and responses to treatment can be easily monitored at a chair-side providing a comfortable dental experience to the patient.

However, the clinical value further relies on the discovery of new information, any alternative therapies, cost-effectiveness, and the safety profile of the recently designed test protocol; consequently, although this POC testing is technically feasible, actual clinical application is still a challenge, and thus, with respect to findings of this study, much further research and investigation are important to validate the biomarkers (IL-17, IL-10) with large populations that suitably account for diversity such as those related to race, region, gender, and age since careful analysis is mandatory before adopting a newly emerged diagnostic test in the current clinical protocol.

## Conclusion

Within the limitations of this study, the findings suggest that IL-17 and IL-10 could discriminate periodontal health from smoker and nonsmoker's periodontitis; however, much further investigations for validity and reliability are essential.
